# Double positivity for rheumatoid factor and anti-CCP autoantibodies: improving referral from primary care of patients suspected of having rheumatoid arthritis

**DOI:** 10.1017/S1463423623000695

**Published:** 2024-01-17

**Authors:** Maria Salinas, Álvaro Blasco, Emilio Flores, Mauricio Minguez, Carlos Leiva-Salinas

**Affiliations:** 1 Clinical Laboratory, Hospital Universitario de San Juan, San Juan de Alicante, Alicante, Spain; 2 Department of Clinical Medicine, Universidad Miguel Hernandez, Elche, Spain; 3 Department of Rheumatology, Hospital Universitario de San Juan, San Juan de Alicante, Alicante, Spain; 4 Department of Radiology, University of Missouri, Columbia, MO, USA

**Keywords:** CCP testing, early diagnosis, laboratory interventions, primary care, rheumatoid arthritis

## Abstract

**Background:**

Rheumatoid arthritis (RA) is a chronic progressive autoimmune inflammatory disease with significant morbidity and mortality. The course of the disease can be modified if diagnosis is early and treatment appropriate.

**Aim:**

In this study, we aimed to evaluate a new strategy for early identification of RA patients in primary care settings (the ‘diagnostic bottleneck’) based on serological biomarkers and to manage inappropriate rheumatoid factor (RF) laboratory test requests.

**Method:**

A two-arm study was carried out. The first arm corresponded to a retrospective observational descriptive study of patients referred for RF testing from primary care using the current laboratory workflow. The second arm included the following prospective interventions: cancelation of RF requests corresponding to patients with previous negative results for RF over a one-year period; and automatic reflex testing antibodies against cyclic citrullinated proteins (anti-CCP) for patients displaying RF values >30 IU/ml. Outcomes from both arms were then compared.

**Findings:**

As double positivity for RF and anti-CCP notably increases the positive likelihood ratio of RA. The intervention enabled a reduction of 2813 tests in 22 months. Moreover, the frequency of unnecessary referrals was reduced from 22% to 8.2%, while that of missed patients decreased slightly (from 21% to 16%), with the number of patients diagnosed per RF request remaining unchanged. In terms of costs, we saved 19.4 RF tests per anti-CCP test added.

We developed a simple and cost-effective strategy for reducing the time to diagnosis of RA that can improve patients’ quality of life. This approach was supported by primary and specialised care.

## Introduction

Rheumatoid arthritis (RA) is a chronic progressive autoimmune inflammatory disorder of unknown etiology that leads to irreversible joint damage and is associated with significant morbidity and mortality (American College of Rheumatology Subcommittee on Rheumatoid Arthritis Guidelines, 2002; Littlejohn and Monrad, 2018). The prevalence of RA, which increases with age, is estimated to be approximately 0.5%–1% in the adult population. Recent studies suggest that more than 2.3 million individuals are diagnosed with RA in Europe, generating annual direct and indirect management costs of over €45 billion (Lundkvist *et al.*, 2008).

The initial presenting features of early RA do not differ substantially from other forms of inflammatory arthritis. The initial manifestations are very varied, and a wide range of conditions that can mimic RA make early diagnosis of RA challenging (Suresh, 2004).

Early identification and treatment of RA can affect disease course, prevent development of joint erosion, and/or delay progression of erosive disease. An early, accurate diagnosis is essential, largely because with RA, there is an ideal window of opportunity for initiation of treatment to slow disease progression and prevent joint damage. Essentially, the earlier treatment is started, the better the outlook for the patient. In fact, RA can be considered a potentially curable condition if identified and treated before it progresses from inflammatory arthritis to established disease (Heidari, 2011).

Serology plays an important role in the diagnosis of RA, and according to the 2010 Classification Criteria of the American College of Rheumatology, at least one positive serology test for rheumatoid factor (RF) and/or antibodies against cyclic citrullinated proteins (anti-CCP) is needed for classification (Aletaha *et al.*, 2010). Anti-CCP are highly specific for RA and are detected in 60%–70% of RA patients; RF is also present in nearly 70% of patients with RA, although it is less specific, with positivity also reported in the healthy population (Ingegnoli *et al.*, 2013). Thus, the combination of both biomarkers significantly increases the positive likelihood ratio of RA. Moreover, evidence suggests that the development of anti-CCP and RF precedes the development of RA (Kokkonen *et al.*, 2011; Lingampalli *et al.*, 2018) and that at early stages, double-positive patients will evolve faster to clinically active RA (Lingampalli *et al.*, 2018).

The need for early diagnosis of RA highlights the importance of adequate management of the disease by primary care physicians (PCPs) (Rat *et al.*, 2004), who evaluate patients at the initial stages. RF testing is frequently requested by PCPs in our area (located on the mid-Mediterranean coast of the Spain and covering mainly mid-size urban areas) although in most cases, positive results are not handled appropriately owing to the low specificity of the marker. Consequently, referral to secondary care is not optimised, thus delaying the diagnosis of RA patients and increasing the number of referrals of healthy patients. Likewise, RA diagnostic delays due to inefficient referral have been described as a main concern widely, and digital diagnostic assessment has been proposed as a valuable tool to advance towards RA early diagnosis (Knitza Johannes and Knevel., 2020; Knevel *et al.*, 2022). Besides the potential of digital tools, as the first-line setting for screening, primary care centres need new, experience-based strategies to improve management of patients suspected of having RA, focusing on streamlining of RF testing and on the criteria for referral of patients with positive results, both of which remain unmet needs.

We believe that defining criteria for testing for RF and anti-CCP (reflex) together in patients with positive results will optimise referral to the rheumatology department. Similarly, early detection of RA will be more frequent, with the consequent positive impact on quality of life.

Our study aims to evaluate a new strategy for early identification of RA patients in primary care settings (the ‘diagnostic bottleneck’) based on serological biomarkers and to manage inappropriate RF laboratory test requests.

## Material and methods

### Study design

We performed a cohort study at San Juan University Hospital (Alicante, Spain). The hospital has 370 beds, serves a population of 234 551 inhabitants in an urban–rural setting, and has nine primary care centres assigned to it, with their respective auxiliary offices.

First, we analysed trends in RF testing by PCPs. All RF tests requested over a 10-year period were counted, and the monthly ratio of RF to creatinine requests (RF/CREA) was also calculated.

Then, to evaluate the impact of our strategy for optimising management of RA from primary care, we defined two arms:

1. Retrospective arm – no intervention: Observational retrospective arm running from 1 January 2017 to 31 March 2019.

This arm included all patients with RF tests ordered by PCPs during the above-mentioned period. Clinical records corresponding to positive patients (RF > 30 IU/ml) were reviewed, focusing on referral to rheumatology and whether a diagnosis of RA was confirmed during the study period. RF > 30 IU/ml (the upper value of our reference interval) was established as the cut-off to enhance sensitivity in our screening approach.

The number of RF-positive patients was counted. From these, we determined the number of patients referred to rheumatology, and from these, we determined the number of patients eventually diagnosed with RA, differentiating between newly diagnosed patients and previously diagnosed patients.

In January 2021, we revisited the clinical records of patients not referred during the inclusion period and recorded the number of cases of RA diagnosed.

1. Prospective arm – Intervention: Observational prospective arm running from April 2019 to January 2021.

In April 2019, a new protocol was established to streamline RF testing and for management of suspected RA in primary care centres in our catchment area. The protocol was designed according our procedure for managing inappropriate laboratory test requests (Salinas *et al.*, 2016) and approved by the Rheumatology Department, primary care specialists, and the clinical laboratory, before being implemented for automation in the laboratory information system.

This intervention included the following:

1. Cancelation of RF requests from primary care and corresponding to patients with previous negative results for RF in a one-year period.

It was also agreed that PCPs could always reorder the canceled RF request if this was clinically justified.

1. Reflex anti-CCP testing:

Automatic reflex testing of anti-CCP IgG for patients with RF > 30 IU/ml and the recommendation, through a comment in the laboratory report, for referral to rheumatology in cases of anti-CCP IgG > 40 IU/ml (positive).

Data were recorded from all patients for whom RF testing was ordered by PCPs during the inclusion period following the procedure detailed for the retrospective arm. However, in this case, we also quantified the number of double positives and differentiated between single-positive and double-positive RA patients.

### Data analysis

To compare the clinical performance of both study arms, we evaluated the following parameters:

- Initial request for RF testing by PCPs*: Number of requests received by the clinical laboratory from primary care.
- Saved tests: Number of RF requests canceled according to the intervention protocol.
- Final RF test: Percentage of RF tests eventually performed from the total requested by primary care.
- Positive RF sample vs. total determinations: Percentage of samples displaying positive results from the total number of samples analysed.
- Anti-CCP IgG tests added: Number of anti-CCP tests added.
- Patients referred to rheumatology: Single-positive and double-positive patients referred to rheumatology once serology results were available.
- New diagnosis of RA: Number of patients diagnosed with RA for the first time after referral.
- Delayed diagnosis of RA: Number of patients with a final diagnosis of RA who were not referred once serology results became available and were diagnosed with RA at a later stage.
- Unnecessary referrals: Number of referrals corresponding to non-RA patients.
- Diagnosed patients per RF test: Number of cases of RA diagnosed per positive RF test.
- Total tests (RF + anti-CCP): Number of canceled RF determinations, subtracting the number of anti-CCP tests added.
*RF test request was adjusted to creatinine tests. Since creatinine is constantly requested in primary care, the calculation of RF to creatinine test request ensures that any variations in a particular test demand are not caused by variations in lab test demand.

The study was approved by the Hospital Research Committee.

### Analytical methods

Anti-CCP were determined using EliA CCP (Thermo Fisher Scientific, Freiburg, Germany) performed in a Phadia 250 laboratory system (Thermo Fisher Scientific, Uppsala, Sweden) according to the manufacturer’s specifications.

RF was determined using turbidimetry with an RF test (Roche, Basel, Switzerland) performed in a COBAS 8000 laboratory system (Roche, Basel, Switzerland) according to the manufacturer’s specifications.

## Results

### Trends in requests for RF testing by PCPs

**Figure 1.**
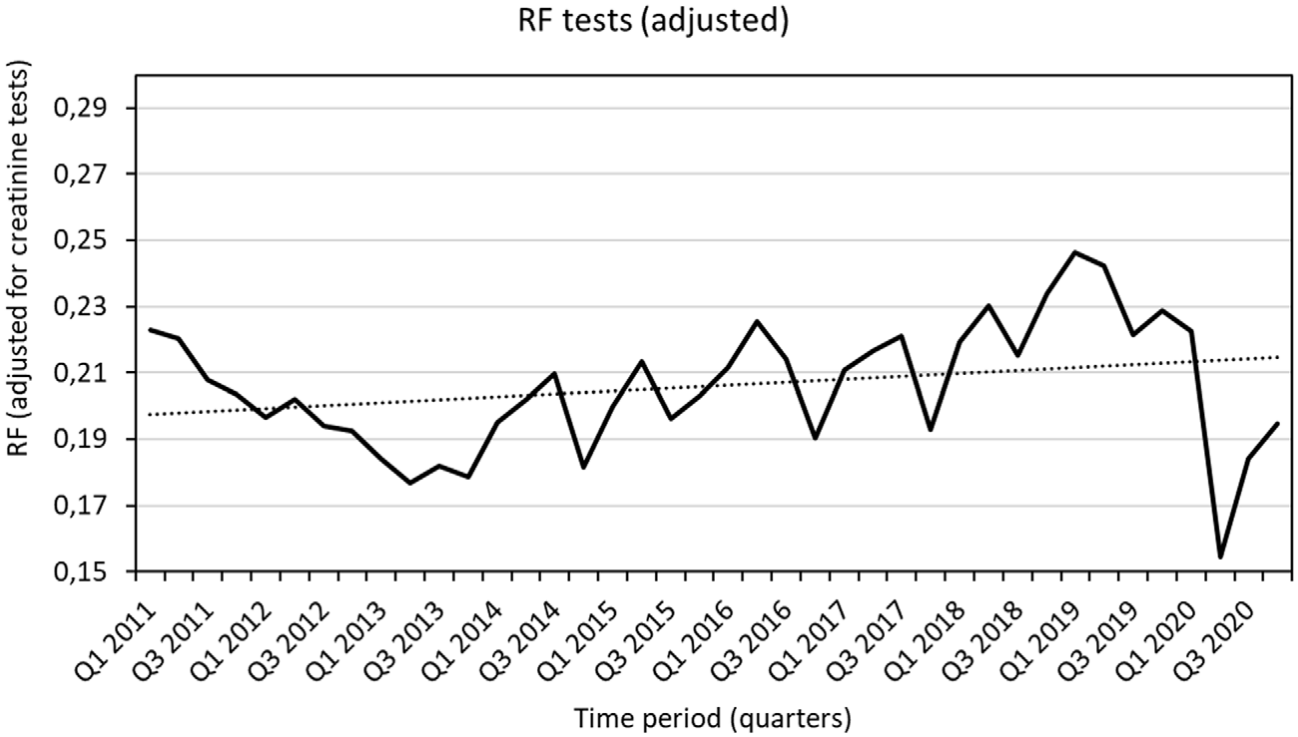
Rheumatoid factor tests ordered by general practitioners from 2011 to 2020 per quarter. Data are shown as adjusted values vs. creatinine requests. RF, rheumatoid factor

From 2011 on, our laboratory registered an increase in requests for RF testing from primary care. In the first quarter of 2019, we reached a peak of 15 812 requests, that is, significantly higher than the 11 995 we performed in 2011. This finding cannot be explained by the general increase in laboratory testing, as observed through the RF/CREA result (Fig. 1). This long-term upward trend was interrupted by the COVID-19 outbreak, although it does indicate that RF is an important and widely used tool in primary care; in fact, PCPs request, on average, one RF determination per every 15 patients selected for blood testing.

### Retrospective arm: classic diagnostic approach (no intervention)

During the 27-month inclusion period, 20% of blood analysis requests from PCPs included RF, that is, 34 308 requests. Of these, 361 sera (1%) revealed results >30 IU/ml and were considered positive, although only 120 patients were referred for specialised evaluation. The 120 patients were selected at the discretion of the PCP, without following a specific protocol, with the associated risk to overlook truly RA patients but also patients with other autoimmune diseases (systemic lupus erythematosus, Sjogren’s syndrome, ANCA-associated vasculitis, autoimmune hepatitis, etc.). Forty of the 120 patients were diagnosed with RA; of these 40 patients, 30 were new diagnoses. Of the 241 patients who remained at the primary care level during the inclusion period, 11 were finally diagnosed with RA.

### Prospective arm: new diagnostic approach (intervention)

During the 22-month inclusion period, 14% of blood analysis requests from PCPs included RF, that is 17 938 requests, from which 2813 were automatically rejected by the system, resulting in a final total of 15 125 RF tests. Of these, 145 sera (0.95%) displayed results >30 IU/ml and were considered positive, and 32 also showed anti-CCP >40 IU/ml and were considered double positives. All the cases involved (double positives) were automatically referred to rheumatology for evaluation. Of these 32 patients, 20 were diagnosed with RA, with 14 of them being diagnosed for the first time. Out of the 138 patients with positive RF but negative anti-CCP results, 20 were subsequently referred to rheumatology; 4 were eventually diagnosed with RA, with 2 being new cases.

**Figure 2.**
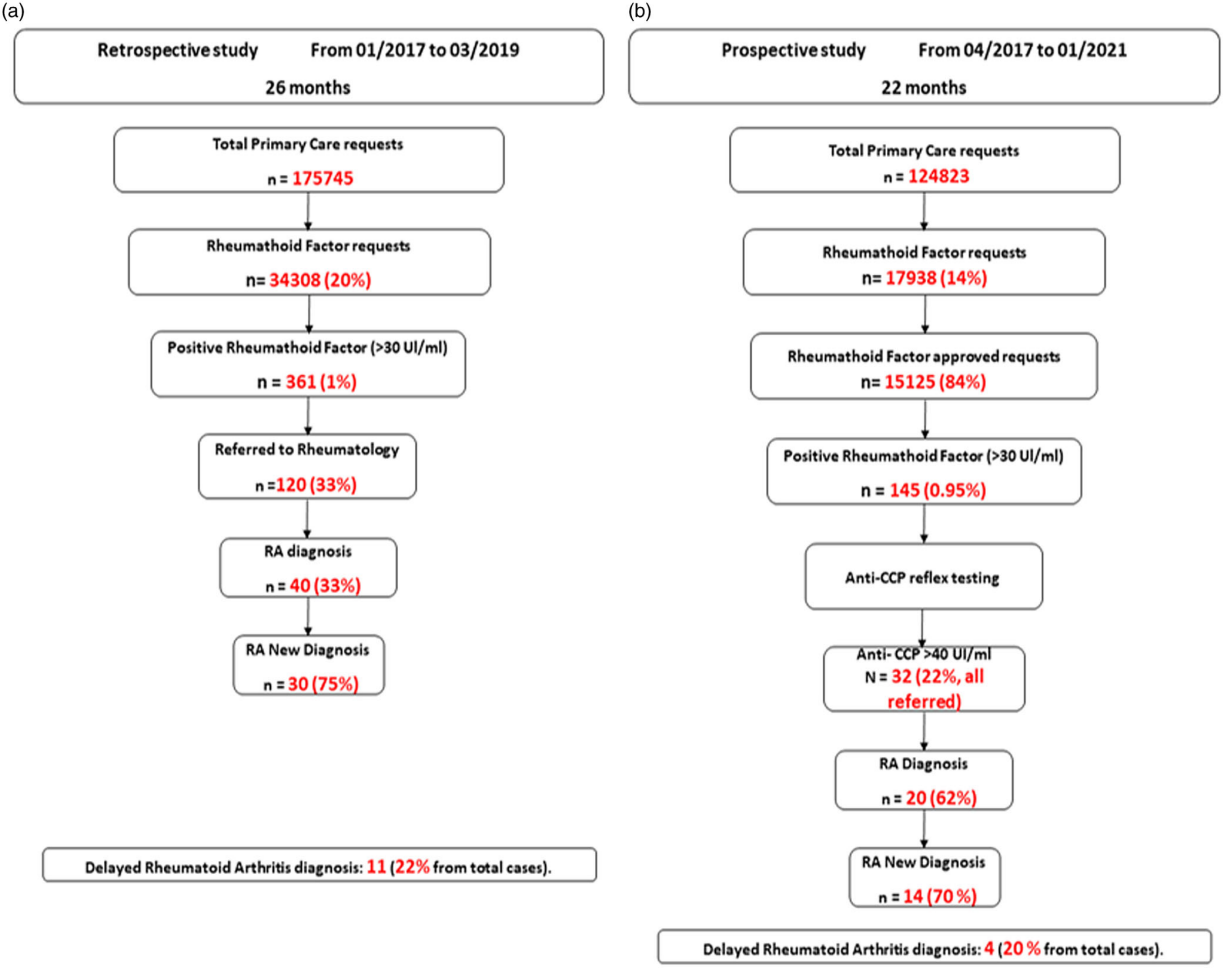
Overview of the analysis according to the two diagnostic approaches for patients suspected of having RA in primary care. (a) Classic diagnostic algorithm (retrospective analysis). (b) Post-intervention diagnostic algorithm (prospective analysis). RF, rheumatoid factor; RA, rheumatoid arthritis

A parallel comparison of both diagnostic algorithms used in this manuscript is outlined in Fig. 2.

### Comparison of the two arms

The intervention strategy enabled a reduction of 2813 tests in 22 months. Moreover, unnecessary referrals were reduced from 22% to 8.2%, while the percentage of missed patients was reduced slightly (from 21% to 16%), and the number of diagnosed patients per RF request was increased (Table 1). In terms of costs, we saved 19.4 RF tests per anti-CCP test added. This finding translates directly into important savings for the clinical laboratory, as test prices differ little, regardless of the commercial platform used for each one. We should also take into account the indirect costs saved as a result of the reduction in referrals.

**Table 1.** Comparison of the results from both diagnostic approaches

	Retrospective study (01/2017 to 03/2019)	Prospective study (04/2019 to 01/ 2021)
Initial RF request by PCPs	34 308	17 938
Tests saved	0	2813
Final RF determinations (% of initial requests)	34 308 (100%)	15 125 (84%)
Positive RF sample vs. total determinations (n, %)	361 (1.05%)	145 (0.095%)
Anti-CCP IgG tests added	0	145
Patients referred to rheumatology	120	32
New diagnosis of RA	30	14
Delayed diagnosis of RA (% total RA patients)	11 (27%)	4 (20%)
Unnecessary referrals	22%	8.2%
Diagnosed patients per RF test	0.11%	0.13%
Total tests (RF + anti-CCP balance)		–2668

PCP = primary care physician; RF = rheumatoid factor; RA = rheumatoid arthritis.

The difference in the % of RF tests from the total requests (20% in the retrospective arm and 14% in the prospective arm) was due COVID lockdown. During lockdown, just urgent diseases were attended by PCPs, and main analytical request was related to COVID-19, and this is the reason behind the decrease in RF test versus the number of blood test request.

## Discussion

We found that our strategy for selecting RA patients for referral successfully improved clinical outcomes from the preinterventional period while reducing the number of tests ordered and the number of referrals.

Our data suggest that the addition of anti-CCP testing in RF-positive patients revealed a primary care population with a higher pretest probability of RA, thus decreasing the number of unnecessary referrals. The ratio of diagnoses of RA per RF test also increased, and the number of patients whose diagnosis was delayed decreased, with fewer false-negative patients in the intervention arm.

The increase in RF testing over time shows that GPs should be instructed to ask for RF testing only in cases of articular symptoms in which RA is a differential diagnosis. The cost impact of adding anti-CCP testing was considerably mitigated by the reduction in RF testing achieved through our intervention. None of the RF tests rejected were reordered by PCPs during the study period. Moreover, indirect savings from the reduction in unnecessary referrals should also be taken into account.

Various publications agree that RA treatment is delayed mainly owing to the delay in referral from PCPs to secondary care and that interventions coordinated between all the healthcare professionals involved in management of RA are needed to overcome this issue, which negatively impacts patient’s quality of life (Rat *et al.*, 2004; Barhamain *et al.*, 2017; Chilton *et al.*, 2021).

Anti-CCP autoantibodies are part of the 2010 ACR/EULAR classification criteria for RA (Aletaha *et al.*, 2010), and evidence has highlighted the added value – in terms of highest probability of true positivity – of the combination of anti-CCP and RF for diagnosis of RA (Infantino *et al.*, 2014; Sun *et al.*, 2014). However, at least in Spain, anti-CCP testing is not frequently ordered from primary care.

As a research group focused on the optimisation of laboratory requests from primary care, we have extensive experience in setting up protocols in agreement with primary and secondary care to improve the cost-effectiveness of diagnosis in various disease areas (Salinas *et al.*, 2013; Salinas *et al.*, 2014; Salinas *et al.*, 2020). We observed a clear need for an intervention in the diagnosis of RA. Our results aim to guide PCPs who are not optimising the tools at their disposal to improve management of RA, with emphasis on early diagnosis. Two simple additional steps, streamlining requests for RF testing and addition of anti-CCP testing, make all the difference.

Our strategy has been based on the increase of specificity associated with double positivity (RF and anti-CCP), but that can still be improved. Verheul and colleagues have proposed that triple positivity (RF, anti-CCP, and anti-CarP) confer even higher specificity (98%-100%) (Verheul *et al.*, 2018). Thus, the triple evaluation approach would be the next step. However, at this moment, is still far from the clinical daily practice. However, positivity for RF and negativity for CCP could still be important for other autoimmune diseases.

Our study has some limitations. The periods compared are not parallel, with the result that ratios were used to increase the comparability of the results, and we did not include data on the average lag times between the first visit to primary care and diagnosis of RA, as the prospective study was biased by the effect of the COVID-19 outbreak, which collapsed the healthcare system. Nevertheless, we do know that double-positive patients from the intervention arm were referred immediately after the blood test, although we do not know the time between availability of positive RF results and referral in the retrospective arm. In any case, it seems that, at best, they were referred as quickly as in the intervention arm.

Another limitation is that our intervention does not prevent missing RA patients being exclusively positive for CCP. According to a systematic review, single positivity to CCP is more common than RF positivity in early RA patients (Whiting *et al.*, 2010). Therefore, CCP testing positioning will be reevaluated following appropriate PCP training on the benefits of CCP testing by using the current algorithm. Thus, in specific settings with appropriate knowledge on RA, CCP testing could be the optimal approach for first-line screening. Also, the addition to the algorithm of other features like tender or swollen joints should be considered (Villeneuve *et al.*, 2013). Likewise, even though we demonstrated that our intervention improved early diagnosis in RF-positive patients, the overall improvement would come from RF-negative and CCP-positive patients who are discarded with in our workflow proposal. Finally, we would want to emphasise that, according to our prior experience, no RA patients are lost if we disregard RF testing for patients who tested negative the year prior (data not shown).

Considering the overall discussion, one can conclude that the ideal first-line screening approach would be both RF and CCP testing. However, the high risk of overtesting from PCPs must be considered, along with the corresponding challenge from an economical perspective.

The data we report should encourage the performance of multicentre studies to evaluate in depth the advantages of anti-CCP testing in primary care and thus improve the diagnosis of this burdensome disease.

## Conclusions

We present an easily applied, cost-effective strategy for improving the diagnosis of RA at the point where the main bottleneck arises, namely, primary care. We recommend joint intervention by laboratory specialists, PCPs, and rheumatologists.
